# Fish Waste Based Lipopeptide Production and the Potential Application as a Bio-Dispersant for Oil Spill Control

**DOI:** 10.3389/fbioe.2020.00734

**Published:** 2020-07-03

**Authors:** Zhiwen Zhu, Baiyu Zhang, Qinhong Cai, Jingjing Ling, Kenneth Lee, Bing Chen

**Affiliations:** ^1^NRPOP Laboratory, Faculty of Engineering and Applied Science, Memorial University of Newfoundland, St. John’s, NL, Canada; ^2^Biotechnology Research Institute of the National Research Council of Canada, Montreal, QC, Canada; ^3^Ecosystem Science Aquatic, Fisheries and Oceans Canada, Ottawa, ON, Canada

**Keywords:** lipopeptide biosurfactant, bio-dispersant, biotechnology, oil spill response, waste management

## Abstract

There is a growing acceptance worldwide for the application of dispersants as a marine oil spill response strategy. The development of more effective dispersants with less toxicity and higher biodegradability would be a step forward in improving public acceptance and regulatory approvals for their use. By applying advances in environmental biotechnology, a bio-dispersant agent with a lipopeptide biosurfactant produced by *Bacillus subtilis* N3-1P as the key component was formulated in this study. The economic feasibility of producing biosurfactant (a high-added-value bioproduct) from fish waste-based peptone as a nutrient substrate was evaluated. Protein hydrolyzate was prepared from cod liver and head wastes obtained from fish processing facilities. Hydrolysis conditions (i.e., time, temperature, pH and enzyme to substrate level) for preparing protein hydrolyzates were optimized by response surface methodology using a factorial design. The critical micelle dilution (CMD) value for biosurfactant produced from the fish liver and head waste generated peptones was 54.72 and 47.59 CMD, respectively. Biosurfactant product generated by fish liver peptone had a low critical micelle concentration of 0.18 g L^–1^ and could reduce the surface tension of distilled water to 27.9 mN/m. Structure characterization proved that the generated biosurfactant product belongs to the lipopeptide class. An alternative to the key surfactant dioctyl sulfosuccinate sodium (DOSS) used in Corexit 9500 has been proposed based on a binary mixture of lipopeptides and DOSS that exhibited synergistic effects. Using the standard baffled flask test, a high dispersion efficiency of 76.8% for Alaska North Slope oil was achieved at a biodispersant composition of 80/20 (v/v) of lipopeptides/DOSS. The results show that fish waste can be utilized to produce a more effective, environmentally acceptable and cost-efficient biodispersant that can be applied to oil spills in the marine environment.

## Introduction

Dispersants have been widely used as a response option to combat oil spills in the marine environment. They are believed to be the most effective way to accelerate the biodegradation of oil at sea (Rongsayamanont et al., 2017). The chemical surfactant in a dispersant facilitates the breakup of oil slicks into small oil droplets, which then are rapidly diluted within the water column to low concentrations (below toxicity threshold limits) that can be readily biodegraded. Dispersant application could prevent shoreline contamination from large oil slicks. This option is appealing when other response options are unavailable due to weather conditions or hard to access locations (e.g., ice covered regions, deep water environments) (Lewis and Prince, 2018).

Following the Deepwater Horizon oil spill incident, over 2.1 million gallons of the chemical dispersant Corexit 9500 was applied at the surface from ships and aircraft, and at the point source of the release by sub-surface injection (Kujawinski et al., 2011). Dioctyl sodium sulfosuccinate (DOSS), one of the key surfactants used in Corexit 9500, was extensively used as a “biomarker” for tracking the transportation and fate of Corexit 9500 owing to its conservative properties and slow biodegradation rate (Dasgupta et al., 2018). This environmental persistence, and knowledge that dispersants increased the bioavailability of the residual oil raised public concerns over the potential environmental impacts associated with dispersant use (Frometa et al., 2017). Strict governmental legislations and growing ecological awareness are thus calling for the development of dispersants of low toxicity and high efficiency. To address this issue, biosurfactant based dispersant formulations have been proposed as a promising alternative to chemical surfactant based products with limited studies in the field (Freitas et al., 2016; Shah et al., 2019).

Biosurfactants are natural surface-active molecules produced by microorganisms during their growth (Zhu et al., 2019). Like the chemical equivalents, they can reduce surface and interfacial tensions, but with lower toxicity and higher biodegradability (Hsu and Nacu, 2003). They also have other desirable features, such as high specificity and strong effectiveness at extreme temperature, salinity and pH conditions (Pacwa-Plociniczak et al., 2011). Lipopeptides, originally identified as secondary metabolites produced by *Bacillus subtilis*, are one group of the most effective biosurfactants reported to date (Chen et al., 2017). An ecotoxicological risk assessment confirmed that lipopeptides have lower toxicity compared to synthetic surfactants, such as sodium dodecyl sulfate (SDS), alcohol ethoxysulphates (AES) and Triton X-100, on various organisms (Santos et al., 2018). Nevertheless, only a few lipopeptides are commercialized due to their high production costs. Around 10–30% of the total biosurfactant production cost arises from the raw material, and up to 60% of the cost was due to the purification process (Mukherjee et al., 2006). To decrease the cost and facilitate biosurfactant based biotechnology development, efforts have thus been devoted to the identification of suitable organic waste materials that can be used for their production.

The seafood industry is of key importance to Canada. Up to 30–80% of the fish body weight is discarded as solid wastes by industrial fish processing operations, posing significant environmental and health problems (Deepika and Manuel, 2014). On the other hand, enzymatic hydrolysis of fish waste can generate peptones that are rich in nutrient and hydrocarbon content (Kristinsson and Rasco, 2000). These peptones are ideal for use as nutrient additives for microbial growth (Vázquez et al., 2008; Safari et al., 2012). Attempts to explore the use of fish peptones as nutrient additives for microbial growth have been reported (Vázquez et al., 2008; Safari et al., 2012). Biosurfactant production with fish waste peptones exhibits great potential in production cost reduction yet limited exploited.

The hydrolysis processes (e.g., the degree of hydrolysis and the composition of peptones) are affected by the composition of the waste material, temperature, hydrolysis time, and the enzyme dose (Bhaskar et al., 2008). Response surface design (RSM) is an experimental based statistical approach to explore the relationships between several factors and assess their effects on one or several desired outputs. RSM has proved to be successful in process optimization (Bhaskar et al., 2008; Gebreluel et al., 2019). The identification and optimization of the waste hydrolysis conditions are important to a cost-competitive biosurfactant production process. Therefore, an in-depth investigation of the optimization of enzymatic hydrolysis with RSM is desired.

In this study, central composite design (CCD), an RSM based methodology was employed to evaluate the multifactor interactions during hydrolysis of fish waste and obtain desired hydrolysis conditions. The generated fish peptones were used as unconventional substrates for biosurfactant production using several *Bacillus subtilis* strains. The biosurfactant production was evaluated using critical micelle dilution (CMD) and surface tension (ST). The critical micelle concentration (CMC), emulsification activity and stability of the selected biosurfactant product were determined. The biosurfactant product was further characterized using Fourier Transform Infrared (FTIR) spectroscopy and matrix-assisted laser desorption/ionization-time of flight- mass spectrometry (MALDI-TOF-MS). Finally, the generated lipopeptide biosurfactant was used to form a dispersant in a mixture with DOSS and its performance on dispersing Alaska North Slope (ANS) crude oil was evaluated.

## Materials and Methods

### Materials

Samples of cod liver and head wastes were from fish processing plants in Newfoundland and Labrador, Canada. Each sample was minced twice using a food processor at medium speed for 120 s. Fresh cod liver and cod head samples were taken for proximate composition analysis prior to immediate storage at −20°C for subsequent experimentation. The proximate composition of fish peptone was determined as follows: the ash content was determined by AOAC 942.05 (AOAC, 2005) and the crude protein was measured by AOAC 2001.11 (AOAC, 2005), with results illustrated in Supplementary Table S1.

Alcalase^®^ 2.4L (endoproteinase from *Bacillus licheniformis*) (Sigma-Aldrich, Canada) was selected as the hydrolysis enzyme. DOSS (C_20_H_37_NaO_7_S), an anionic surfactant due to the charged oxygen atom at the head of the surfactant (Steffy et al., 2011), used as the co-surfactant for dispersant formation was purchased from Sigma-Aldrich. The molecular weight of DOSS is 444.56 g/mol. ANS crude blends from ExxonMobil was used for evaluation of dispersant effectiveness.

### Optimization of Enzymatic Hydrolysis

To generate fish peptone for biosurfactant production, enzyme hydrolysis was performed. The hydrolysis conditions were optimized by employing the RSM with CCD. Four independent variables [i.e., temperature (A, °C), hydrolysis time (B, h), enzyme doze (C, %v/w) and different fish wastes (D)] were examined. The final response was defined as the degree of hydrolysis (DH). All the experimental runs were separated into three blocks to waive the effects of testing facility. The parameters, levels and sequences of experimental treatments are summarized in Table 1.

**TABLE 1:** Central composite design of fish waste hydrolysis.

**Block**	**Time (h)**	**Enzyme dose (%v/w)**	**T (°C)**	**Fish waste**	**Block**	**Time (h)**	**Enzyme dose (%v/w)**	**T (°C)**	**Fish waste**
Block 3	3	2	45	Head	Block 3	3	2	45	Liver
Block 3	3	2	45	Head	Block 3	3	2	45	Liver
Block 1	2	1	50	Head	Block 1	2	1	50	Liver
Block 2	4	1	50	Head	Block 2	4	1	50	Liver
Block 2	2	3	50	Head	Block 2	2	3	50	Liver
Block 1	4	3	50	Head	Block 1	4	3	50	Liver
Block 3	3	0	55	Head	Block 3	3	0	55	Liver
Block 3	3	0	55	Head	Block 3	3	0	55	Liver
Block 3	1	2	55	Head	Block 3	1	2	55	Liver
Block 3	1	2	55	Head	Block 3	1	2	55	Liver
Block 1	3	2	55	Head	Block 1	3	2	55	Liver
Block 1	3	2	55	Head	Block 1	3	2	55	Liver
Block 2	3	2	55	Head	Block 2	3	2	55	Liver
Block 2	3	2	55	Head	Block 2	3	2	55	Liver
Block 3	3	2	55	Head	Block 3	3	2	55	Liver
Block 3	3	2	55	Head	Block 3	3	2	55	Liver
Block 3	5	2	55	Head	Block 3	5	2	55	Liver
Block 3	5	2	55	Head	Block 3	5	2	55	Liver
Block 3	3	4	55	Head	Block 3	3	4	55	Liver
Block 3	3	4	55	Head	Block 3	3	4	55	Liver
Block 2	2	1	60	Head	Block 2	2	1	60	Liver
Block 1	4	1	60	Head	Block 1	4	1	60	Liver
Block 1	2	3	60	Head	Block 1	2	3	60	Liver
Block 2	4	3	60	Head	Block 2	4	3	60	Liver
Block 3	3	2	65	Head	Block 3	3	2	65	Liver
Block 3	3	2	65	Head	Block 3	3	2	65	Liver

**FIGURE 1:**
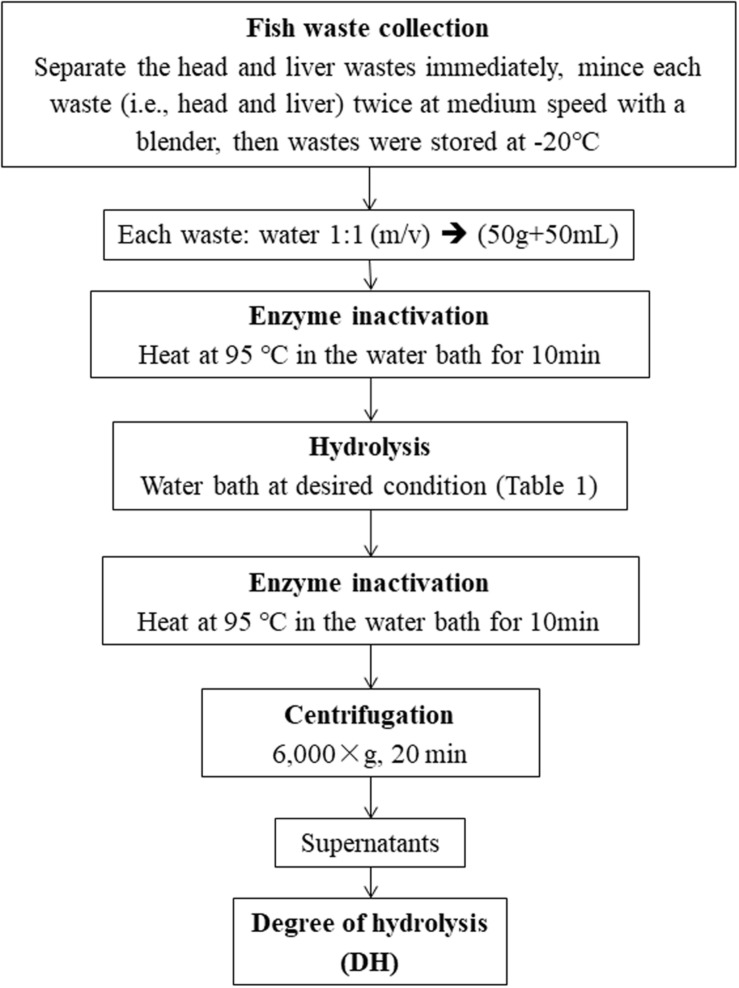
Flow chart of the enzyme hydrolysis process.

The experimental procedures for enzymatic hydrolysis are illustrated in Figure 1. For each experimental run, 50 g of waste sample was added into a 125 mL Erlenmeyer flask and mixed with equal volumes (50 mL) of distilled water (1:1 w/v). The flask was then heated in a water bath at 90°C for 10 min to deactivate any endogenous enzymes in the fish wastes. Then, Alcalase was added to the flask with designed enzyme dose. The enzyme hydrolysis of this experimental run was performed at the given hydrolysis temperature and time (Table 1). When this run was terminated, the Alcalase in the flask was desaturated by heating at 95°C in a water bath for 10 min. The mixture in the flask was then centrifuged at 6,000 rpm for 20 min. The supernatant was collected for the DH measurement.

The DH was estimated using the trichloroacetic acid (TCA) method (Hoyle and Merritt, 1994). Generally, a 50 mL hydrolyzate sample was mixed with 50 mL 20% TCA to generate a 10% TCA solution. This solution was centrifuged at 10,000 rpm to separate the TCA-soluble and TCA-insoluble hydrolyzates. The supernatants with 10% TCA soluble hydrolyzates were analyzed for nitrogen by the macro-Kjeldahl method (AOAC, 2005). The DH was calculated as:

(1) $$D\,H=\frac{10\%\,T\,C\,A-s\,o\,l\,u\,b\,l\,e\,\,N\,\,i\,n\,\,s\,a\,m\,p\,l\,e}{T\,o\,t\,a\,l\,N\,i\,n\,s\,a\,m\,p\,l\,e}\times 100\%$$

Based on the results of system optimization, the desired set of hydrolysis condition was adopted to produce fish liver (FL) and fish head (FH) peptones. The total carbon content, total organic carbon content and total nitrogen content were determined by Shimadzu Total Organic Carbon/Total Nitrogen analyzer (Model: TOC-VCPH).

### Biosurfactant Producing Microorganisms

Two types of fish peptones generated in section “Optimization of Enzymatic Hydrolysis” were evaluated as a nutrient substrate for biosurfactant production. The biosurfactant producers used in this study were screened from oil-contaminated seawater samples in the Atlantic Ocean by the Northern Region Persistent Organic Pollution Control (NRPOP) laboratory (Cai et al., 2014). Among the screened *Bacillus* strains, *Bacillus subtilis* N3-1P, N3-4P, and N2-6P were identified as promising and economic lipopeptide producers (Cai et al., 2014). The commercialized lipopeptide production strain *Bacillus subtilis* 21332 was also selected in this research for performance comparison.

The preparation of the seed culture followed the method described by Zhu et al. (2016). The inoculation broth was prepared by autoclaving (121°C for 20 min) a mixture comprised of 8.0 g of BD Difco^TM^ Nutrient Broth 23400 (Fisher Scientific Company, Ottawa, Canada) and 3.5 g of NaCl in 1 L of distilled water. For each inoculum, a loopful of the selected *Bacillus subtilis* strain colony was transferred into a 125 mL Erlenmeyer flask containing 50 ml inoculum broth. The flask was incubated in a rotatory shaker at 200 rpm under 30°C. After 24 h, the *Bacillus* strain in this flask was used as inoculum at the 2% (v/v) level.

### Biosurfactant Production Using Fish Peptones

#### Biosurfactant Production

To evaluate the feasibility of fish waste based peptones as a biosurfactant production medium, we tested three different fermentation scenarios: (1) fish waste based peptone as a carbon source; (2) fish waste based peptone as a nitrogen source, and (3) fish waste based peptone as a comprehensive substrate. The conventional biosurfactant production medium for selected *Bacillus* strains was collected from the previous study (Zhu et al., 2016). This medium was comprised of carbon and nitrogen sources, the supplemented mineral salts, and trace elements. Glycerol (10 g L^–1^) and NH_4_SO_4_ (10 g L^–1^) were used as the carbon and nitrogen sources, respectively. The composition of the supplemented mineral salts was (g L^–1^): NaCl (15); FeSO_4_⋅7H_2_O (2.8 × 10^–4^); KH_2_PO_4_ (3.4); K_2_HPO_4_⋅3H_2_O (4.4) and MgSO_4_⋅7H_2_O (1.02). The composition of the trace element solution was as follows (g L^–1^): ZnSO_4_ (0.29); CaCl_2_ (0.24); CuSO_4_ (0.25); and MnSO_4_ (0.17). The trace element solution was sterilized separately and then applied at 0.5 ml L^–1^ of distilled water.

##### Carbon substitution medium

Each of the fish peptones (i.e., FH and FL peptones) was evaluated as the carbon source for biosurfactant production. Glycerol (10 g L^–1^) used as the carbon source in the conventional medium was replaced by the FH or FL peptones, at a concentration of 10 g L^–1^. The composition of the supplemented mineral salts and trace elements remains the same as the conventional biosurfactant production medium.

##### Nitrogen substitution medium

Each of the fish peptones (i.e., FH and FL peptones) was evaluated as the nitrogen source for biosurfactant production. NH_4_SO_4_ (10 g L^–1^) used as the nitrogen source in the conventional medium was replaced by the FH or FL peptones, at a concentration of 10 g L^–1^. The composition of the supplemented mineral salts and trace elements remains the same as the conventional biosurfactant production medium.

##### Alternative comprehensive medium

Each of the fish peptones (i.e., FH and FL peptones) was evaluated as a comprehensive biosurfactant production medium for further cost reduction. *Bacillus* strains (i.e., *Bacillus subtilis* N3-1P and *Bacillus subtilis* 21332) that can use fish peptones as carbon and nitrogen sources were selected in this study. This comprehensive medium contains one fish peptone and key supplement minerals. Each of FH and FL peptones was added into distilled water at a series of concentrations (g L^–1^): 10, 20, 30, 40, and 60. Key supplement minerals were added as follows (g L^–1^): FeSO_4_⋅7H_2_O (2.8 × 10^–4^) and MnSO_4_ (0.17).

##### Biosurfactant production

Three types of fish waste based mediums were prepared separately for biosurfactant production. The conventional biosurfactant production medium was used as a control. Fifteen milliliters of each culture medium was added into a 50 mL Erlenmeyer flask. Each selected *Bacillus* strain inoculum was inoculated into the flask at a ratio of 2% (v/v) and incubated in a shaking incubator (200 rpm) at 30°C for 7 days. After incubation, the culture broth was centrifuged at 6,000 g for 15 min. The cell-free broth was then collected. Biosurfactant production was evaluated with ST, emulsification index (EI_24_) and CMD values. All experimental runs were performed in triplicate.

#### Production Evaluation

##### Surface tension

Surface tension was measured by the ring method using a Du Nouy Tensiometer (CSC Scientific). Fifteen-milliliter liquid was subjected to the determination of ST in a petri dish. To ensure the reliability of tested results, the average of three independent measurements was taken.

##### Critical micelle dilution

Critical micelle dilution indicates the concentration of biosurfactants in the medium. It corresponds to the dilution of this medium required to reach its CMC (Shavandi et al., 2011). CMD was determined following the method described by Cai et al. (2017). In general, each cell-free culture broth was diluted with distilled water at different ratios and subjected to ST measurement. The dilution process stopped when the ST exceeded 40 mN/m, and the dilution ratio was recorded as the CMD for this culture broth. All the measurements were performed in triplicate.

##### EI_24_

The EI_24_ of a culture broth was determined by the addition of 2 mL culture aliquot to 2 mL hexadecane and vortexed for 2 min to create an optimum emulsion. Each test was performed in triplicate.

(2) $$E\,I_{24}=\frac{t\,h\,e\,h\,e\,i\,g\,h\,t\,o\,f\,t\,h\,e\,e\,m\,u\,l\,s\,i\,f\,i\,e\,d\,l\,a\,y\,e\,r}{t\,h\,e\,h\,e\,i\,g\,h\,t\,o\,f\,t\,h\,e\,t\,o\,t\,a\,l\,l\,i\,q\,u\,i\,d\,p\,h\,a\,s\,e}\times 100\%$$

By repeating the reading after 24 h, an indication of the stability of the emulsions is obtained. EI_24_ = 0% indicates no emulsification and El_24_ = l00% means 100% emulsification.

### Characterization of Generated Biosurfactants

To reduce the biosurfactant production cost, the *Bacillus* strain generated in the laboratory that can grow in a comprehensive medium with the highest CMD value was selected for product characterization. *Bacillus subtilis* N3-1P led to the highest biosurfactant production (in terms of CMD) in FH and FL based comprehensive medium and thus was selected for further biosurfactant production and characterization. Two biosurfactant products were purified with organic solvents and then subjected to the characterization of physical-chemical properties, including their STs, CMC values and stabilities. The chemical structures of two biosurfactant products were determined by FTIR and MODI-TOF-MS.

#### Biosurfactant Purification and CMC Determination

*Bacillus subtilis* N3-1P was inoculated separately into 30 g L^–1^ FL based comprehensive medium and 30 g L^–1^ FH based comprehensive medium following the method described in section “Biosurfactant Production.” After incubation, each culture broth was centrifuged at 12,000 × *g* for 10 min. The cell-free supernatant was then adjusted to pH 2.0 with HCl and stored overnight at 4°C. The sediment was then harvested by centrifuging at 12,000 × *g* for 10 min. The acidified biosurfactant pellet was dissolved again into 100 mL distilled water. Sodium hydroxide was added to adjust the pH value to 7. The biosurfactant product was recovered with organic solvent extraction. An equal volume of chloroform-methanol solution (2:1 v/v) was used to extract the target biosurfactant products. The bottom layer with the target biosurfactant product was collected, and the organic solvent was removed by rotary evaporation.

CMC is defined as the surfactant concentration necessary to initiate micelle formation. The CMCs of two biosurfactant products were determined. To achieve the CMC value of one biosurfactant product, the ST was plotted as a function of biosurfactant concentration. In this plot, two straight lines were then extrapolated from the concentration-dependent and concentration-independent sections and intersected (Supplementary Figure S1). A tangent to this plot was constructed from this intersection point A. The intersection point B of the tangent and the plot was the CMC value of this biosurfactant product (Sheppard and Mulligan, 1987; de Oliveira et al., 2013). The lowest ST of each biosurfactant product was recorded to reflect the surface tension reduction ability.

#### Stability Analysis

The stability of two generated biosurfactant products was evaluated as changes in the surface activities in response to environmental factors (i.e., salinity, pH, and temperature). For each biosurfactant product, a stock solution was prepared at a biosurfactant concentration of 1 CMC. To investigate the stability of biosurfactant at various salinities, NaCl was added into the stock solution to achieve the desired salinity (i.e., 1, 2, 3, and 4%). The pH effect was determined by adjusting the pH value (2, 4, 6, 8, 10, and 12) of the stock solution using 1 N NaOH or 1 N HCl. The heat stability of this biosurfactant product was evaluated by incubated each stock solution at the desired temperature (i.e., 0, 25, 50, 75, and 100°C) for 120 min. Fifteen milliliters of each biosurfactant solution at a designed environmental condition were collected and subjected to ST test.

#### Composition Analysis

The composition of two biosurfactant products was analyzed using the thin layer chromatography (TLC) analysis. Each biosurfactant product was dissolved in 1 mL of methanol and analyzed on TLC silica gel plates (Sigma Aldrich). The developing solvent used for the chromatography was chloroform: methanol: acetic acid (60:25:5, v/v). The spots on a TLC plate were visualized with standard spray reagent as follows:

(1) The amino acid content could be visualized as a dark purple color when sprayed with ninhydrin reagent and then heated at 105°C for 5 min.
(2) The sugar content on the plate could be spotted as a dark orange or brown color when sprayed with phenol-sulfochromic acid and heated at 105°C for 5 min.
(3) The plate was inserted into an iodine chamber for the characterization of lipid-containing spots (purple color).

#### Structural Analysis

##### FTIR Spectroscopy

A purified biosurfactant product was directly characterized by FTIR-attenuated total reflection (FTIR-ATR) spectroscopy (Bruker Tensor). The spectral measurement was performed in the transmittance mode. IR was traced over the range of 400–4000 cm^–1^. All data were corrected for the background spectrum.

##### MALDI-TOF-MS

The chemical structure of each FL or FH based biosurfactant product was examined by a SCIEX MALDI TOF/TOF System. Each purified biosurfactant product was dissolved into 10 mL distilled water and then passed through a 0.2 μm filter before the test. For mass spectrometric analysis of the isolated lipopeptide biosurfactant, a 2 μL portion of biosurfactant solution was mixed with an equal volume of matrix medium [a saturated solution of α-cyano-4-hydroxycinnamic acid in 50% aqueous acetonitrile containing 0.1% (v/v) trifluoroacetic acid]. The positive-ion detection and reflector mode were used. The acceleration and reflector voltages were 20 and 23.4 kV, respectively, in the pulsed ion extraction mode. Postsource decay mass spectra was obtained with the same sample to increase tolerance to contamination and specificity of the analysis.

### Biodispersant Generation and Performance Evaluation

#### Lipopeptide and DOSS Based Biodispersants

The lipopeptide product was used as a substitution to chemical surfactant DOSS. The FL based lipopeptide product had a lower CMC value (0.18 g L^–1^) than the FH based lipopeptide and thus was selected for biodispersant preparation. The dispersant generation process was modified from the methodology developed by Shah et al. (2019). Synthetic seawater was prepared by adding 3.5 g sea salt into 100 mL distilled water. The biosurfactant stock solution (solution A) was prepared at a concentration of 20 CMC (3.6 g L^–1^), and the DOSS surfactant solution was directly used as stock solution B. A series of co-surfactants were prepared by mixing stock solution A and B at a ratio of 0:10; 2:8; 4:6; 6:4; 8:2; and 10:0 (v/v). Polyethylene glycol 400 (PEG 400) was selected as the solvent for biodispersant preparation given its low toxicity and a good performance in previous biodispersant synthesis studies (Zhang et al., 2018). Each emulsion was prepared by adding ANS crude oil (0.1 mL) to synthetic seawater (1 mL) and then mixed well with 0.1 mL of a co-surfactant solution. Finally, 0.2 mL PEG 400 was added as a solvent. The changes in the emulsification process over a period of time (10 and 30 min) were observed visually and recorded with images to evaluate the stability of the emulsion solution.

#### Interactions Between Lipopeptide and DOSS

The lipopeptide-DOSS interaction in each binary mixture system was evaluated with the system CMC values, as equation (3) indicates (Shah et al., 2019). The molar mass of lipopeptide and DOSS was 1049 g/mol (Rongsayamanont et al., 2017) and 444.56 g/mol, respectively. The CMC value for DOSS is 0.6 mM.

(3) $$\frac{1}{C\,M\,C^{*}}=\frac{\alpha }{C\,M\,C_{l\,i\,p\,o\,p\,e\,p\,t\,i\,d\,e}}+\frac{1-\alpha }{C\,M\,C_{D\,O\,S\,S}}$$

α indicates the mole fraction of lipopeptide.

### Evaluation of Biodispersant Effectiveness

Each biodispersant formulated with co-surfactants and solvent was further evaluated for the dispersant effectiveness. The co-surfactants were prepared with biosurfactant stock solution A (20 CMC, 3.6 g L^–1^) and DOSS solution B at ratios of 0:10; 2:8; 4:6; 6:4; 8:2; and 10:0 (v/v). PEG 400 was selected as the solvent. Each co-surfactant solution was mixed with a solvent at a ratio of 3:7 (v/v) to form a biodispersant. The dispersant effectiveness was evaluated using the baffled flask test (BFT) at a dispersant to oil ratio (DOR) of 1:25 (v/v) under 4 and 25°C (Venosa et al., 2002).

For the BFT test, a volume of 120 mL artificial seawater (Instant Ocean sea salt) was added into a baffled flask. Then, 100 μL of ANS oil was dispensed onto the surface of the seawater. Four microliters of a biodispersant were added into the flask to achieve a DOR of 1:25. The flask was then shaken at 200 rpm on the orbital shaker. After 10 min, the flask was removed from the shaker and stood still for another 10 min. The first 2 mL water sample was discarded from the stopcock at the bottom of the flask. Then, 30 mL of water sample was collected and extracted with 5 mL dichloromethane (DCM) (Sigma-Aldrich, ACS reagent) in a separatory funnel. The extraction process was repeated three times and a total of 15 mL DCM was used for the extraction of dispersed oil in the water sample. The experimental run with 100 μL of ANS oil and 120 mL artificial seawater in a baffled flask was used as control. The experimental run with 120 mL artificial seawater only in the baffled flask was performed as blank. The absorbance of each extract was determined at wavelengths of 340, 370, and 400 nm, respectively, using an ultraviolet spectrophotometer. The area under the absorbance vs. wavelength curve between 340 and 400 nm was calculated by the trapezoidal rule using equation (4) (Venosa et al., 2002). The ratio of the areas of the dispersed oil and total oil added into the system is the dispersant efficiency.

(4) $$A\,r\,e\,a=\frac{\left(A\,b\,s_{340}+A\,b\,s_{370}\right)\times 30}{2}+\frac{\left(A\,b\,s_{370}+A\,b\,s_{400}\right)\times 30}{2}$$

### Statistical Analysis

The statistical software Design-Expert^®^ 8.0.6 was used for experimental design, data analysis and result validation. Design at center points in each factorial block, axial points and axial (star) points were performed in duplicate (as Table 1 illustrates). Each experimental design in biosurfactant production, product characterization and BFT studies were all performed in triplicate. Means and standard errors were calculated for pooled results of each design condition. The statistical analysis were analyzed using OriginPro^®^ 9 software package.

## Results and Discussion

### Optimization of Fish Waste Hydrolysis

The influences of hydrolysis time (factor A), enzyme-to-substrate ratio (factor B), temperature (factor C) and waste material (i.e., head and liver) (factor D) on the enzymatic hydrolysis were determined using RSM. Among the four independent variables, the enzyme-to-substrate ratio (B) (*p* < 0.0001) and hydrolysis temperature (C) (*p* = 0.0004) had a higher impact on the hydrolysis result. The effect of hydrolysis time (A) (p = 0.0132), though less than factors B and C, was also considered to be significant (*p* < 0.05). The impact of waste composition on the final DH results was minor (*p* = 0.6450). The interactions among the different variables were also limited (*p* > 0.05).

**FIGURE 2:**
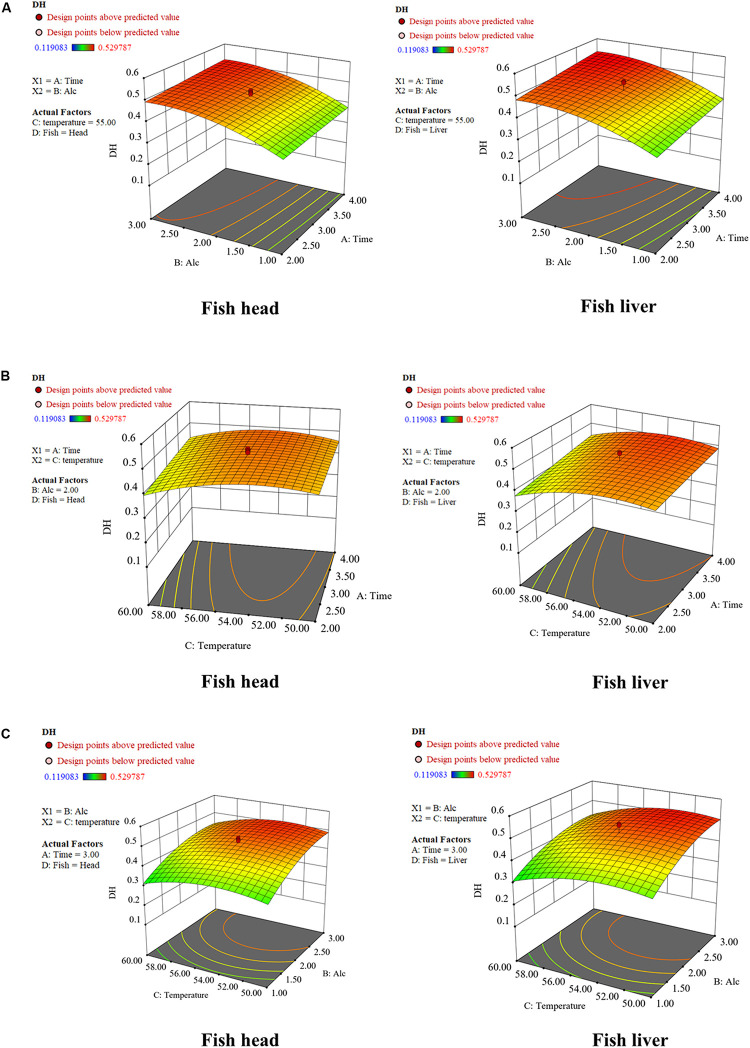
Response surface graphs for degree of hydrolysis (DH) as a function of **(A)** time and enzyme dose; **(B)** temperature and time; **(C)** temperature and enzyme dose. Fifty grams of fish waste sample [fish head (FH) or fish liver (FL)] were hydrolyzed separately by Alcalase^®^ 2.4 L following the experimental conditions listed in Table 1. The hydrolysis conditions were optimized by employing the RSM with Central Composite Design.

The response surface graphs for DH listed in Figure 2 verified the results of the ANOVA analysis. Alcalase was employed in previous fish hydrolysis studies because of its high enzyme activity. Hydrolyzation can be achieved in a relatively short time under moderate conditions (Benhabiles et al., 2012). In this study, Alcalase was found to possess broad specificity to achieve a high DH. The DH of both waste materials had a positive response to the enzyme-to-substrate ratio. The optimized enzyme-to-substrate ratios were estimated at 2.72% for fish liver and 2.92% for fish head (Table 2). A continuous increase of enzyme dose could further improve the DH of fish waste, however, at a slower rate. The Catla (*Catla catla*) hydrolysis study conducted by Bhaskar and Mahendrakar (2008) drew the same conclusion. The growth of DH rate slowed down with an increase in Alcalase dose.

**TABLE 2:** Optimization of fish waste hydrolysis.

**Fish waste**	**Time (h)**	**Alcalase (%)**	**Temperature (°C)**	Estimated DH* (%)	**Validated DH (%)**
Liver	4	2.72	52.51	53.39	51.61
Head	4	2.92	54.07	52.35	49.37

*DH, degree of hydrolysis.

The optimized temperatures were estimated at 52.51 and 54.07°C for FL and FH peptone generation, respectively (Table 2). The DH then gradually reduced as temperature increased. It is believed that the Alcalase slowly becomes thermally denatured above 55°C. This result is in accordance with the conclusion drawn by Ovissipour et al. (2009). Though hydrolysis time had less significance than the enzyme dose and hydrolysis temperature, an increase of this factor, could also contribute to a higher DH, as observed in Figure 2. Similar to an increase of enzyme dosage, the prolonged hydrolysis time could further improve DH, though at a slower increase rate.

Following the optimized enzymatic hydrolysis conditions, the verification results are illustrated in Table 3. The verification indicated a good agreement between the experimental results and the RSM models.

**TABLE 3:** Characterization of fish waste generated peptones.

**Peptones**	**Total carbon (mg g^–1^)**	**Total organic carbon (mg g^–1^)**	**Total nitrogen (mg g^–1^)**	**Ash (%)**	**C/N**	**Reference**
Fish head	405.1 ± 3.2	73.4 ± 0.3	98.1 ± 0.7	5.8	4.12	
Fish liver	399.9 ± 2.3	66.2 ± 0.4	128.9 ± 0.8	6.3 ± 0.1	3.1	
Tryptone	N/A	N/A	133	6.6	3.4	Aspmo et al., 2005
Soytone	N/A	N/A	94	12.0	4.4	Aspmo et al., 2005
Yeast extract	N/A	N/A	114	13.1	3.9	Aspmo et al., 2005

*N/A, not available.

The characterization of hydrolyzed peptones is listed in Table 3. As observed, the nitrogen content was 98.14 and 128.92 mg g^–1^ for FH and FL peptones, respectively. These results are comparable to the values found for the widely used commercial peptones (Table 3). Similarly, the C/N ratios for both peptones fell into the range of commercial peptones. FH peptone had a relatively higher C/N ratio than the FL peptone. As observed, both peptones possessed high concentrations of total carbon, total nitrogen and C/N content and thus could be used as good substitutes for traditional biosurfactant production mediums.

### Production of Biosurfactants Using Fish Peptones

**FIGURE 3:**
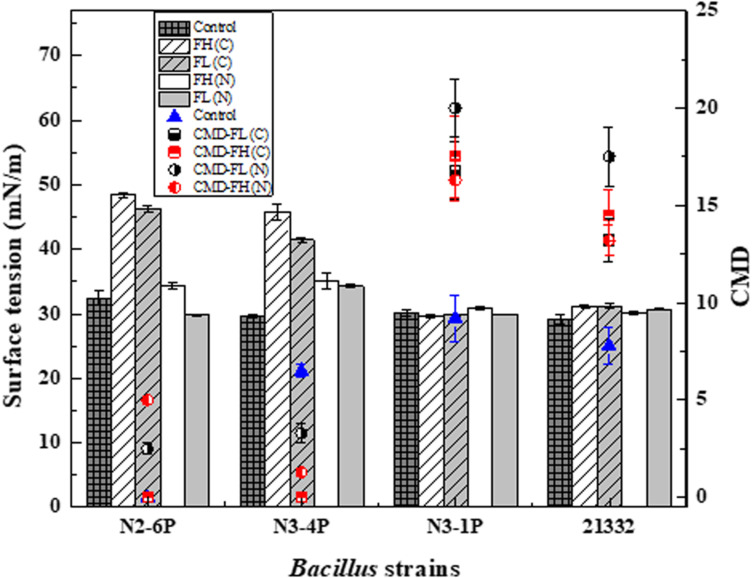
Biosurfactant production in terms of surface tension (ST) and critical micellar dilution (CMD) of *Bacillus subtilis* N2-6P, N3-4P, N3-1P, and 21332 using fish head (FH) and fish liver (FL) peptones as carbon and nitrogen sources. Strains were inoculated at 2% concentration into three types of mineral medium. (1) Control: glycerol (10 g L^– 1^); NH_4_SO_4_ (10 g L^– 1^) as carbon and nitrogen sources; (2) Fish peptones as carbon sources: glycerol were replaced by FH or FL at 10 g L^– 1^ [in terms of FH(C) and FL(C)]; and (3) Fish peptones as nitrogen sources: NH_4_SO_4_ (10 g L^– 1^) was replaced by FH or FL at 10 g L^– 1^ [(in terms of FH(N) and FL(N)]. Strains were incubated at 30°C at 200 rpm for 7 days. Results are expressed as the average ± SD of three independent measurements.

Figure 3 provides the feasibility of using fish peptones to support bacteria growth and biosurfactant production as carbon and/or nitrogen sources. The performance of fish peptones as a medium substitute was assessed based on the degree of ST reduction and the CMD value of the medium. The production and accumulation of biosurfactants reduced the ST of the culture medium. Previous studies proved that biosurfactants can reduce the ST of distilled water from 72 to 27 mN/m (Pacwa-Plociniczak et al., 2011). Continuous biosurfactant production cannot further reduce the ST of culture medium, however, could led to the formation and accumulation of micelles in the medium. This suggests that the dilution of the micelles could be used to reflect biosurfactant yield. Biosurfactant production mediums with high CMD value could hold promise for industrial lipopeptide production, as they maximize the bacterial performance and minimize the production cost.

As shown in Figure 3, when using peptones as the nitrogen source for biosurfactant production, ST reduction was observed in all the samples [i.e., FH (N) and FL (N)]. The ST reduction of the substituted nitrogen medium was comparable to that of the control (29.7 mN/m of the culture medium). *Bacillus subtilis* N3-4P reported a higher ST of the culture medium than the other strains [35.12 and 34.3 mN/m for FH(N) and FL(N), respectively]. FL had a better performance than FH when serving as the nitrogen source. Though all the strains could utilize fish peptones for biosurfactant production, the final yield varied. *Bacillus subtilis* N3-1P and 21332 had better responses to fish peptones than *Bacillus subtilis* N2-6P and N3-4P. *Bacillus subtilis* N3-1P using FL peptone as the nitrogen source had the highest CMD value (20 CMD).

The effect of nitrogen selection on biosurfactant production has been long recognized (Makkar and Cameotra, 2002). Some strains had a better performance with inorganic nitrogen sources (e.g., NH_4_NO_3_, NaNO_3_, etc.). For example, *Bacillus subtilis* MTCC 1427 preferred to use nitrate ions for biosurfactant production (Das and Mukherjee, 2007). Such preference can be explained by the readily available high nitrogen content in the inorganic nitrogen sources. Conversely, *Bacillus subtilis* N3-1P and 21332 exhibited preference for organic nitrogen. Organic nitrogen (e.g., yeast extract or protein hydrolyzates) contains nutrients that are key to cell growth and polysaccharides secretion (Nurfarahin et al., 2018) and is believed to be the inducer to stimulate lipopeptide production (Zhu et al., 2016). Lipopeptide synthesis was directly regulated by non-ribosomal peptide synthetases, which can directly incorporate some amino acid to the final lipopeptide product (Schwarzer et al., 2003). Therefore, the hydrolyzed amino acids in FL samples might be more suitable for biosurfactant production. Interestingly, in our study, the cell-free culture mediums were not able to form emulsions as the control medium did (EI_24_ data not shown). It was assumed that the hydrolyzed medium might inhibit emulsification formation.

On the other hand, those strains had a different response to fish peptones as the carbon source. Biosurfactant production, as reflected by ST reduction, was only observed in *Bacillus subtilis* N3-1P and *Bacillus subtilis* 21332 samples (Figure 3). While using FL(C) as the carbon source, the ST of the culture broth reduced to 29.8mN/m and 31.2 mN/m for *Bacillus subtilis* N3-1P and *Bacillus subtilis* 21332, respectively. Similarly, the ST of FH(C) based culture medium reduced to 29.6 and 31.2 mN/m for *Bacillus subtilis* N3-1P and *Bacillus subtilis* 21332, respectively. *Bacillus subtilis* N3-1P had the highest biosurfactant production [17.5 CMD and 16.8 CMD for FH(C) and FL(C), respectively] when using fish peptones as the carbon substitute.

*Bacillus* strains need to consume organic carbon sources for growth and metabolic activities. However, Pepi et al. (2013) reported that the biosurfactant production process was inhibited when using an animal fat substrate as sole carbon source, owing to the high composition of palmitic acid (26.40%) and oleic acid (24.16%). This could help explain the suppression of biosurfactant production by *Bacillus subtilis* N3-4P and N2-6P in the current study, as those fatty acids have also been widely identified in fish wastes (Khoddami et al., 2009).

**FIGURE 4:**
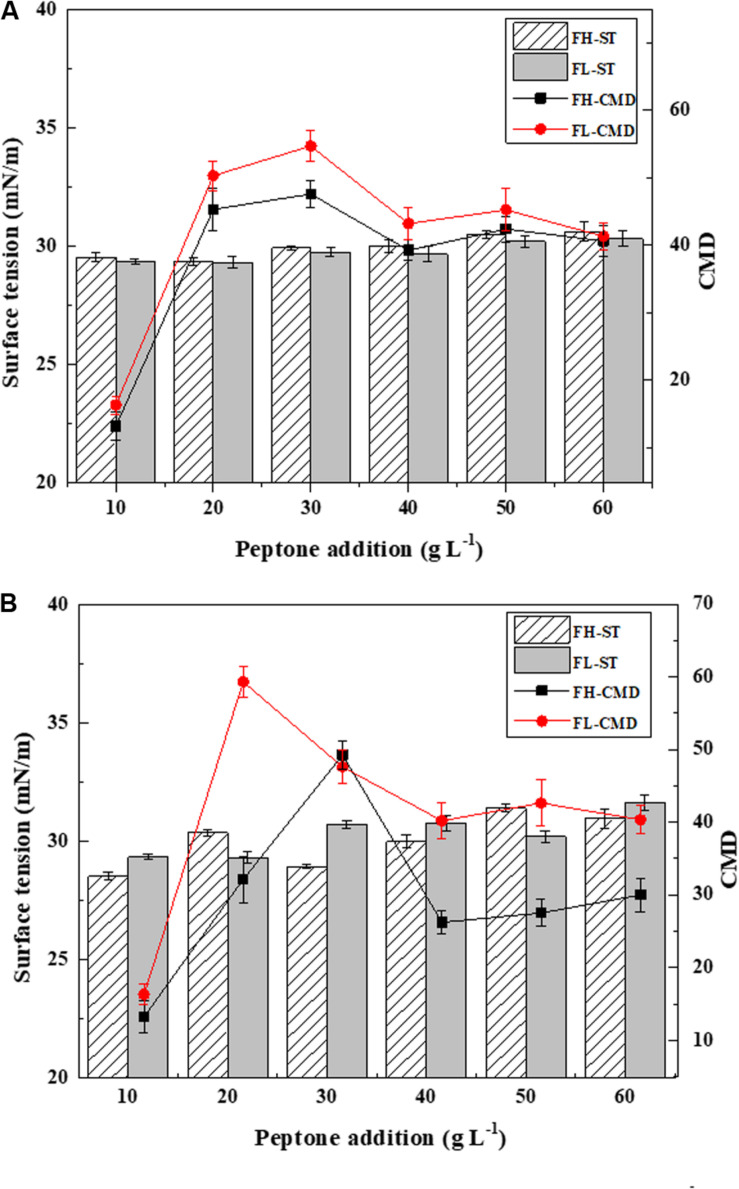
Biosurfactant production (ST and CMD) using fish head (FH) and fish liver (FL) peptones at various concentrations as the comprehensive production medium for **(A)** *Bacillus subtilis* N3-1P; **(B)** *Bacillus subtilis* 21332. Results are expressed as the average ± SD of three independent measurements.

Fish waste peptones were further investigated as comprehensive mediums for biosurfactant production using *Bacillus subtilis* N3-1P and *Bacillus subtilis* 21332. The results are presented in Figure 4. Both strains shared a similar biosurfactant production trend. Higher biosurfactant production rates were obtained when using FL peptone as the sole medium. The highest biosurfactant yields were 54.72 and 59.33 CMD for *Bacillus subtilis* N3-1P and *Bacillus subtilis* 21332, respectively. Using 30 g L^–1^ FH peptone as the growth medium, the CMD values for biosurfactant produced by *Bacillus subtilis* N3-1P and *Bacillus subtilis* 21332 were 47.59 and 49.24 CMD, respectively, These results were much higher compared to the control medium, whose biosurfactant yield was 7.8 and 9.2 CMD for *Bacillus subtilis* N3-1P and *Bacillus subtilis* 21332, respectively. When peptone concentrations higher than 30 g L^–1^, the biosurfactant production was inhibited.

The medium composition is a key factor affecting the structural diversity and yield of biosurfactants. It is believed that the hydrolysis pretreatment greatly increased the bioavailable carbon and nitrogen concentrations, thus stimulating biosurfactant production by *Bacillus subtilis*. On the other hand, the different amino acid composition in FH and FL peptones could contribute to the varied biosurfactant yields in the two peptone mediums. For example, higher concentrations of valine and lysine could dramatically increase biosurfactant production, whereas alanine and arginine could inhibit the production process of *Bacillus* strains (Makkar and Cameotra, 2002). The inhabitation of biosurfactant production at a higher peptone concentration could be explained by the excessive nitrogen in the medium. Reis et al. (2013) believed that insufficient nitrogen facilitates the biosurfactant production process. Under a nitrogen limiting condition, continuous cell growth and dividing are hindered. A microbial metabolism favoring the production of secondary metabolites is then promoted, and the expression of the biosurfactant production gene is stimulated (Nurfarahin et al., 2018).

Al-Wahaibi et al. (2014) determined biosurfactant production by *Bacillus subtilis* B30, using glucose and molasses as carbon sources. The ST of broth medium increased to 40.84 mN/m after dilution for eight times. Joshi et al. (2013) optimized biosurfactant production by *Bacillus licheniformis* R2 in bench-scale bioreactors. Their final CMD value of the generated biosurfactant was 100 CMD after incubation for 96 h. Hydrolysis pretreatment for biosurfactant production acquired increasingly attention. Valenzuela-Ávila et al. (2020) adopted pre-hydrolyzed waste frying oil as a carbon source for *Bacillus subtilis* to produce biosurfactant. After incubation for 120 h, the CMD for culture broth was 1.25 CMD with a final ST of around 40 mN/m. Using the acid hydrolyzate of defatted algal biomass as a sole substrate, the yield of biosurfactant produced by a *Bacillus subtilis* strain reached 80 CMD (Yun et al., 2020). The results generated in this study indicated that fish waste hydrolyzate could serve as a promising substrate for biosurfactant production.

### Characterization of Generated Biosurfactants

The physical-chemical properties of two biosurfactant products generated by *Bacillus subtilis* N3-1P were determined. The CMC values were 0.18 g L^–1^ and 0.3 g L^–1^, respectively, for the crude biosurfactant generated from the FL and FH peptones (Supplementary Figure S2). These values are compatible with the biosurfactant products generated by other *Bacillus subtilis* strains (Das and Mukherjee, 2007; Barros et al., 2008). The biosurfactant products generated from the FL and FH based peptones could reduce the surface tension of water from 72 to 27.9 mN/m and 27.8 mN/m, respectively. It is widely acknowledged that lipopeptides could reduce the surface tension of distilled water to 27–30 mN/m (Das and Mukherjee, 2007; Mnif and Ghribi, 2015). The TLC analysis revealed that two biosurfactant products primarily consisted of lipid and protein.

**FIGURE 5:**
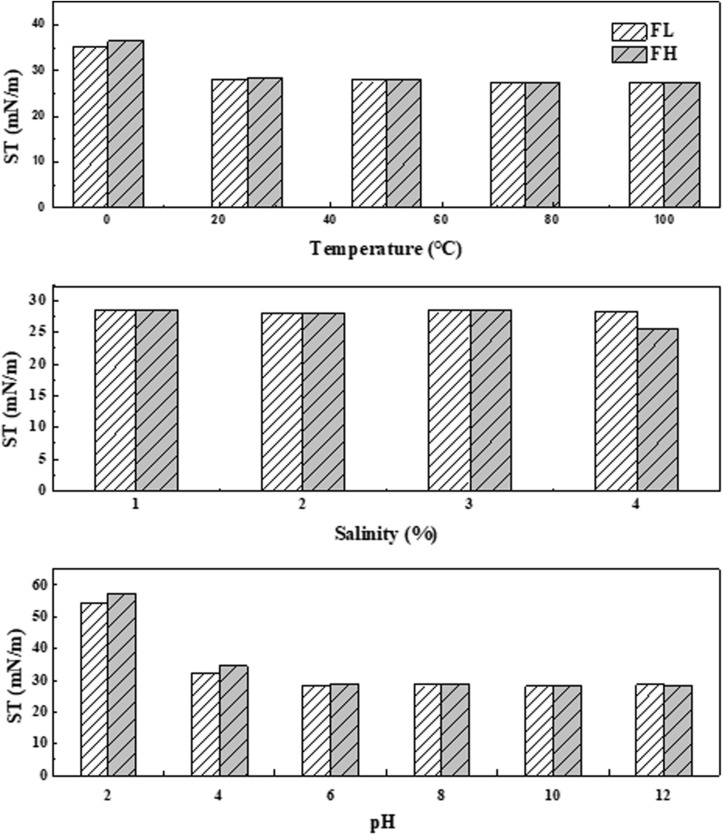
Stability of biosurfactants generated by *Bacillus subtilis* N3-1P using fish liver (FL) or fish head (FH) as a comprehensive production medium at various temperatures, salinity and pH conditions in terms of surface tension (ST).

The stability of two biosurfactant products generated from fish wastes was assessed under a wide range of environmental conditions (i.e., temperature, salinity, and pH). As Figure 5 illustrates, the surface activities of generated biosurfactants were positively correlated with temperature. The surface tensions remained in a narrow window of 35.2 – 27.3 mN/m from 0°C to 100°C. The results proved that both biosurfactants had Krafft temperatures (also known as the critical micelle temperatures) below 0°C. This Krafft point is closely related to their structure (i.e., hydrophilic and lipophilic groups) and ionic character (Nakama, 2017). The observed thermostable nature of produced biosurfactants is consistent with findings from other studies. For example, biosurfactant products produced by four different *Bacilli* isolates could be kept stable for 9 days at 80°C (Joshi et al., 2008). Salinity also had a limited effect on the stability of both generated biosurfactants (Figure 5). This behavior may be predictable since high salt concentrations can considerably reduce the size and shape of the micelle, thus affecting the functional properties of a biosurfactant (Al-Bahry et al., 2013).

It has also been reported that the structure and size of the micelles in a water-oil system could be affected by environmental pH (Das et al., 2009). Gudiña et al. (2010) reported instability under acidic conditions due to the presence of negatively charged groups at the end of biosurfactant molecules. In this study, the ability of the fish waste generated biosurfactant products to reduce surface tension was inhibited at very low pH levels (i.e., pH = 2) (Figure 5) due to the formation of precipitates. However, the surface tensions of fish waste generated biosurfactants remained almost constant at a pH range from 4 to 12. These results highlight the applicability of the crude biosurfactant produced by *Bacillus subtilis* N3-1P in a cold marine environment.

**FIGURE 6:**
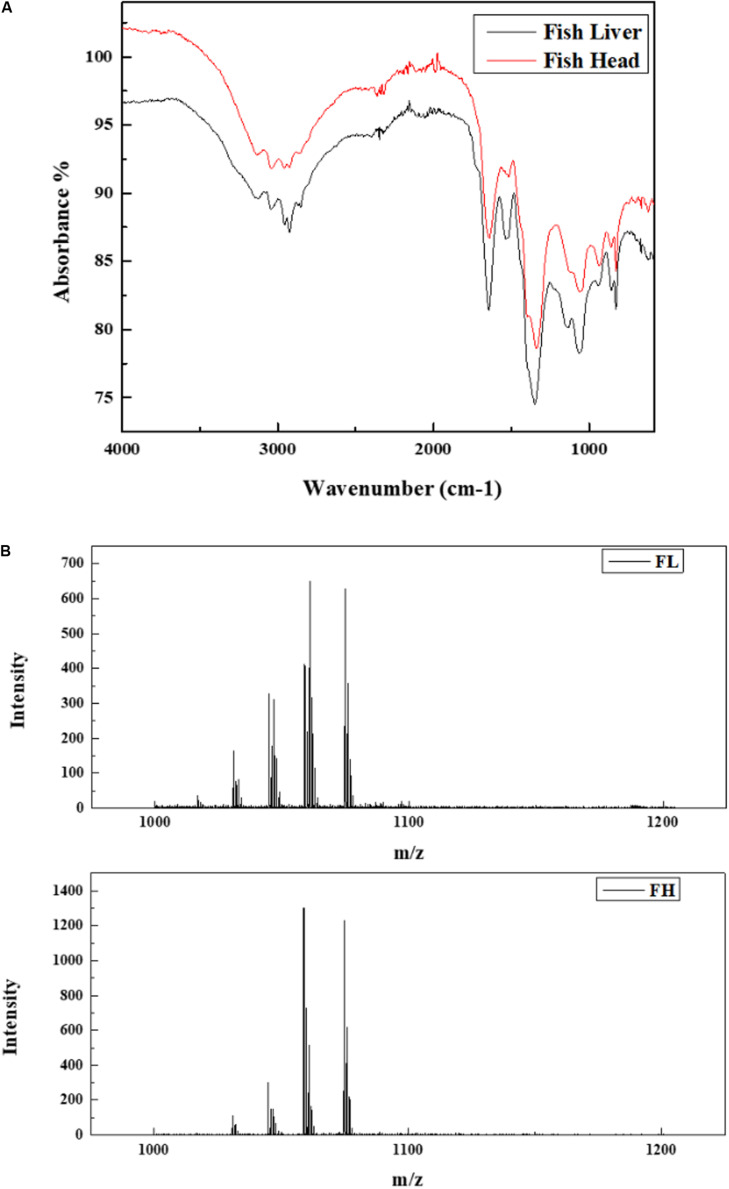
Characterization of biosurfactants generated by *Bacillus subtilis* N3-1P using fish liver (FL) and fish head (FH) as minimum production medium. **(A)** FTIR analysis; **(B)** MALDI-TOF analysis.

The FTIR spectrums were examined to obtain information on the functional groups of two generated biosurfactant products, and the results are illustrated in Figure 6A. The FL and FH based biosurfactants showed an apparent similarity with stretched intense peaks in the region of 500 – 4500 cm^–1^. The stretching absorption between 1050 – 1150 cm^–1^ may denote a C-O stretch and could be primary, secondary or tertiary alcohol. The absorbance peaks at 1350 – 1650 cm^–1^ evidenced the presence of amide groups. Another broad stretched peaks between 2850 and 3050 may be contributed by the -CH_3_, -CH_2_ or -CH groups. The presence of a board O-H band (3300 to 2600 cm^–1^) and the strong C = O stretching (1600-1700 cm^–1^) evidenced the existence of carboxylic acid groups. The FTIR spectrums suggested that the biosurfactant products were lipopeptides.

Figure 6B presents the structure of two generated biosurfactant products analyzed by MALDI-TOF. This result clearly indicates that the two products have a very similar composition. Two groups of lipopeptides, namely surfactin (m/z 1016, 1030, 1044, 1058, and 1060) and iturin (1026, 1043, 1065, 1079), were identified in both fish waste generated biosurfactants.

### Formation of Biodispersants for Oil Dispersion

**FIGURE 7:**
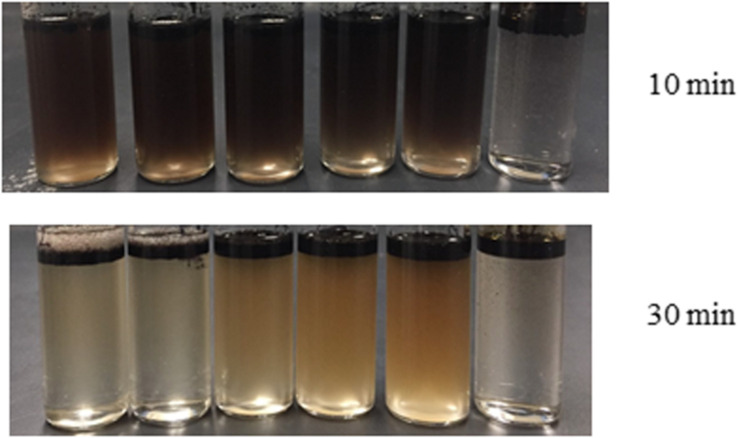
Visualization of emulsion stability (from left to right, biosurfactant concentration increased from 0 to 100%). A series of co-surfactants was prepared by mixing biosurfactant solution (3.6g L^– 1^), and DOSS at a ratio of 0:10; 2:8; 4:6; 6:4; 8:2; and 10:0 (v/v). Each emulsion system contains ANS crude oil (0.1 mL), synthetic seawater (1 mL), co-surfactant (0.1 mL) and polyethylene glycol 400 (0.2 mL). Changes in the emulsification process over a period of time (10 and 30 min) were recorded.

The formation of an oil-in-water emulsion is important for an effective oil dispersion process. Therefore, the emulsion capacity of the biodispersants and their stability were evaluated. As a chemical surfactant of major concern in Corexit 9500, DOSS was gradually replaced with the biosurfactant generated in this study. The ideal CMC values of the lipopeptide-DOSS binary mixture were predicted by the Clint model (Table 4). The addition of lipopeptide rapidly decreased the system CMCs. As Figure 7 indicates, the emulsification ability of co-surfactants was increased with the increase of lipopeptide concentration. At *t* = 10 min, a homogeneous emulsion was rapidly formed after vortexing. However, most of the oil droplets in the vial rose to the top, forming a clear layer of crude oil above the aqueous phase after 30 min. The 8:2 ratio of co-surfactants left the highest content of oil emulsion in the aqueous phase and thus were expected to have a better performance as a dispersant. The result demonstrated that the emulsion formed by co-surfactants had stronger stability than that of a single surfactant. Such a phenomenon also suggested a synergistic interaction between the generated lipopeptide biosurfactants and DOSS during micelle formation. McClements and Jafari (2018) believed surfactants tend to pack between each other at the water-oil interface. Therefore, during the interaction of DOSS and lipopeptide, the attachment and interaction of the hydrophilic heads of two surfactants could affect the optimum curvature of the interface, and reduce the interfacial tension between two medium (Acosta and Bhakta, 2009). The attraction of two surfactants leads to the decrease of system CMCs, and subsequently enhanced emulsion. The existence of electrolyte (i.e., ions in the seawater) in the solution could further enhance such effects (Zhang et al., 2004; Azum et al., 2014).

**TABLE 4:** Critical micelle concentrations (CMC) of lipopeptide-DOSS binary mixture.

**Lipopeptide (%)**	**0**	**20%**	**40%**	**60%**	**80%**	**100%**
Lipopeptide (mL)	0	0.02	0.04	0.06	0.08	0.1
DOSS (mL)	0.1	0.08	0.06	0.04	0.02	0
Mole fraction (α)	0	0.56	0.77	0.88	0.95	1
CMC* of system	0.267	0.21	0.194	0.187	0.182	0.18

CMC*: the ideal CMC value of each binary system.

### Effectiveness of Prepared Biodispersants

**FIGURE 8:**
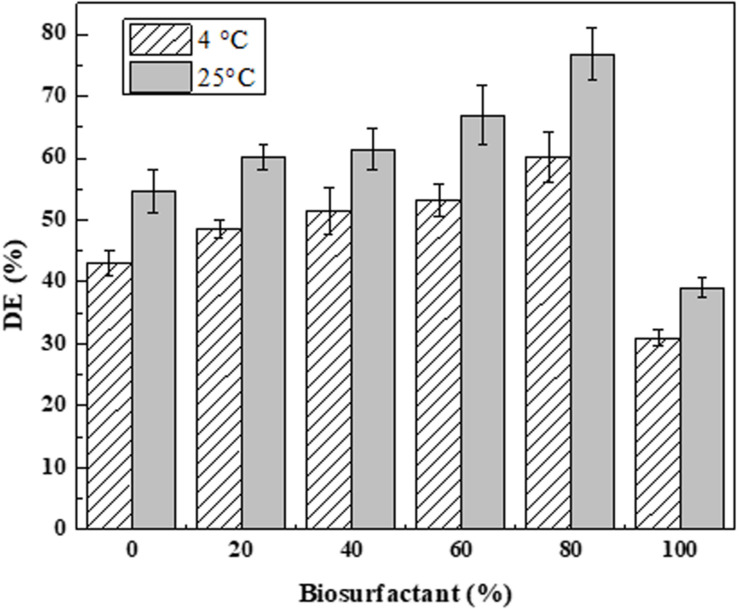
Effectiveness of ANS oil dispersion (DE) by the newly developed dispersant. A series of co-surfactants was prepared by mixing biosurfactant solution (3.6 g L^– 1^), and DOSS at a ratio of 0:10; 2:8; 4:6; 6:4; 8:2; and 10:0 (v/v). Each biodispersant was composed of one co-surfactant and PEG 400 at a ratio of 3:7 (v/v). The effectiveness of biodispersant was determined at a dispersant to oil ratio (DOR) of 1:25 (v/v) under 4 and 25°C following the baffled flask test.

New biodispersants were formulated in this study by gradually replacing DOSS with lipopeptide biosurfactants. Figure 8 presents the dispersion efficiency of prepared biodispersants using lipopeptide-DOSS mixtures at various biosurfactant concentrations (i.e., 0, 20, 40, 60, 80, 100%). The highest dispersion effectiveness of the newly developed biodispersant was 60.2 and 76.8% under 4 and 25°C, respectively. Such dispersion efficiencies were achieved using the 8:2 (v/v) ratio of lipopeptide-DOSS as the key surfactant ingredient in the biodispersant. However, when using 100% fish waste-based biosurfactant product as a key surfactant for biodispersant formulation, the oil dispersion efficiency was less than those composed of co-surfactants. Rongsayamanont et al. (2017) also reported a higher dispersant efficiency using pure lipopeptides than the one reported in this study. Fish peptones could affect the composition of crude lipopeptides products and impact their emulsification and oil dispersion abilities accordingly. The improved dispersant effectiveness was due to the synergistic effect co-surfactants as dispersants formulated with co-surfactants had a better performance than single surfactant. Temperature is an important environmental factor that contributed to dispersant effectiveness. Lower temperature (4°C) resulted in reduced dispersant effectiveness of all the newly developed bio-dispersants. This is in accordance with previous studies as dispersant and oil viscosity can be significantly increased by a decrease in water temperature and consequently, can inhibit the dispersant effectiveness (CEDRE, 2016; Zhang et al., 2018).

### Economic Feasibility of Biodispersant Development

Till now, several biosurfactant products have been commercialized, such as rhamnolipids (AGAE Technologies – United States; Jeneil Biosurfactant – United States; BioFuture – Ireland; TensioGreen – United States), sodium surfactin (KANEKA – Japan and SABO – Italy), and sophorolipids (Synthezyme – United States). Their industrial application, particularly in the petroleum and environmental field, has been reviewed (Silva et al., 2014; Akbari et al., 2018). The retail price for commercialized lipopeptides ranges from US$ 10/mg for pure surfactin (98% purity) (Freitas et al., 2016) to US$629.5/kg (Lotioncrafter, 2020).

There are several options to optimize production and thus reduce the production costs of biosurfactants. Given that raw material accounts for 10–30% of the total biosurfactant production cost (Kosaric and Sukan, 2014), exploration of waste/by-products as substrates for biosurfactant production is an attractive and important option to reduce the production cost (De Almeida et al., 2016). In addition to our study on fish wastes, various waste materials, such as olive mill wastes (Ramírez et al., 2016), sisal pulp hydrolysis (Marin et al., 2015) and defatted algal biomass (Yun et al., 2020), have been pre-hydrolyzed for biosurfactant production. Youssef et al. (2007) produced lipopeptide biosurfactant from a petroleum reservoir, with a reported material cost to produce one mol lipopeptides (∼1 kg) of $22.4. Freitas et al. (2016) estimated the production cost of sophorolipids generated by *Candida bombicola* URM 3718 was around $0.1–0.22/L using sugarcane molasses, corn steep liquor and soy waste as substrate. This cost was much lower than the retail price of sophorolipids, which is $2.5–6.3/kg (Freitas et al., 2016). Biosurfactant productivity could be further improved by the development of effective bioreactors (Zhu et al., 2019), and the design of engineering strains (Banat et al., 2014). In addition, the purification of generated biosurfactant contributes to around 60% of the overall production cost. By using biosurfactant in a crude form, particularly in the environmental field, such purification costs could be avoided.

It is believed that after production optimization, the cost of lipopeptides could be comparable to that of DOSS surfactant (USD$277/kg, Sigma-Aldrich). Therefore, their future application in the environmental and petroleum industry as an environmentally friendly alternative to chemical surfactants could be promising. It is worth mentioning that instead of being sent to landfill where it could impose further costs and environmental pollution (e.g., landfill leachate), the conversion of fish wastes hydrolyzate into high-value biosurfactant products could turn into a new profit stream for fishery industries.

## Conclusion

This study examined the use of fish waste protein hydrolyzates as a low-cost substrate for microbe growth to biosynthesize high-added-value fermentative products, including biosurfactants. The fish waste based medium achieved a higher biosurfactant yield than the one generated by the conventional medium. Moreover, this study showed that emulsion capacity and stability were important to the effectiveness of dispersants. Lipopeptide biosurfactant generated in this study served as an effective alternative to chemical surfactants. The synergistic effect of co-surfactants (DOSS and biosurfactant) could facilitate emulsion formation and marine oil dispersion. Additional work will be needed to further optimize the waste based production medium and evaluate the biosurfactant production kinetics to facilitate their future larger-scale production. The properties of the prepared biodispersants, such as their stability, biodegradability and ecotoxicity, will also need to be systematically evaluated. This study demonstrated an effective approach for generating environmentally friendly dispersants for marine oil spill response over the range of temperatures encountered in the world’s oceans.

## Data Availability Statement

The raw data supporting the conclusions of this article will be made available by the authors, without undue reservation, to any qualified researcher.

## Author Contributions

ZZ and BZ designed the study. ZZ and JL conducted the experiments. ZZ and QC performed the sample collection and analysis. ZZ, BZ, and BC contributed to the data interpretation. ZZ wrote the manuscript. KL contributed to the manuscript preparation and critical revision. All authors contributed to manuscript revision.

## Conflict of Interest

The authors declare that the research was conducted in the absence of any commercial or financial relationships that could be construed as a potential conflict of interest.
