# Supplementary material for: Fish Waste Based Lipopeptide Production and the Potential Application as a Bio-Dispersant for Oil Spill Control
Source: Front Bioeng Biotechnol. 2020 Jul 3;8:734. doi: 10.3389/fbioe.2020.00734 (PMC7347989; doi:10.3389/fbioe.2020.00734)
Supplement: Supplementary file 1 [file Data_Sheet_1.docx]

Figure S1 Methodology for CMC determination


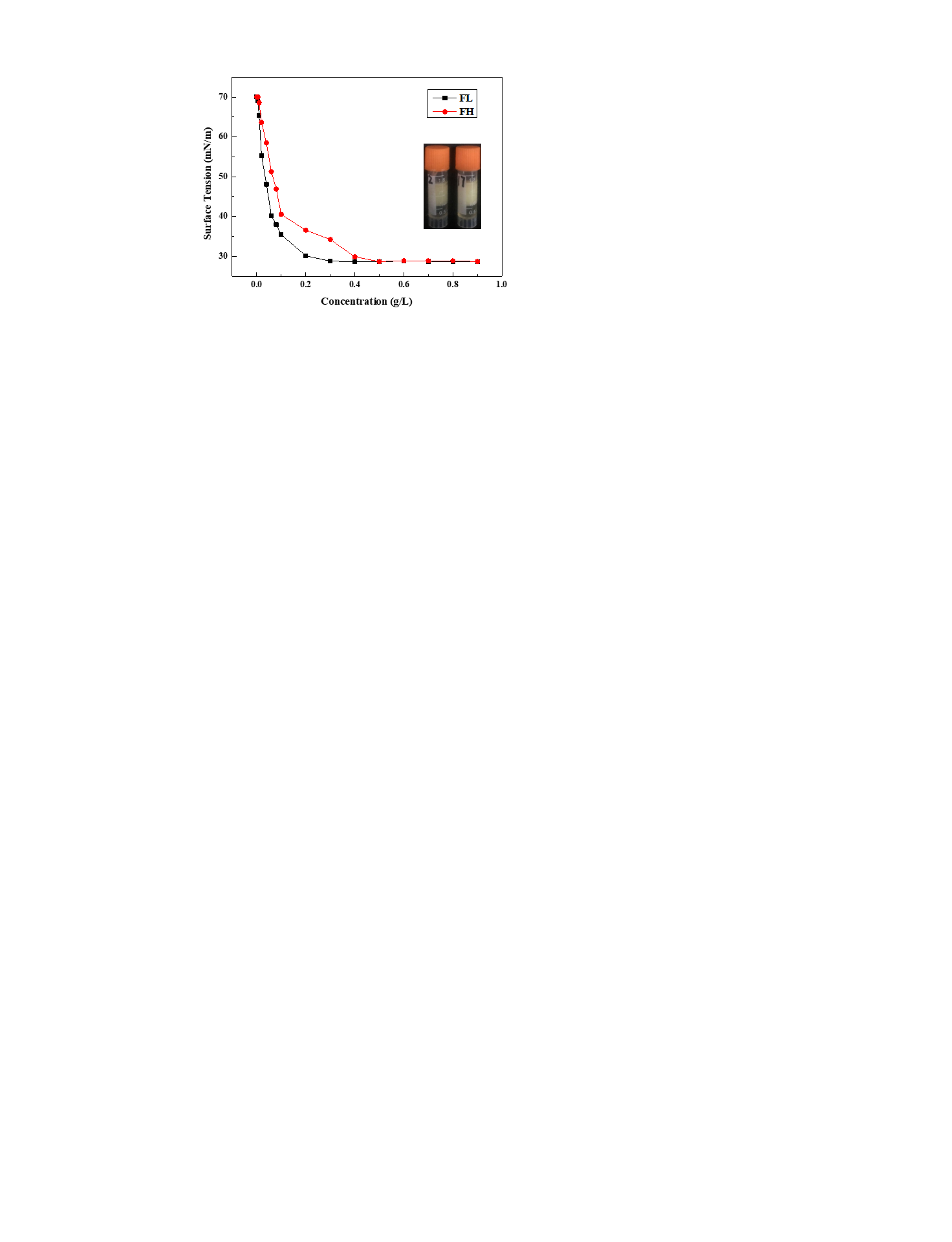


Figure S2 CMC values of fish-waste-based biosurfactants generated by *Bacillus Substilis* N3-1P

Table S1 Proximate composition of fish wastes

| Constituent | Fish Liver | Fish Head |
| --- | --- | --- |
| Moisture (%) | 71.3 | 58.9 |
| Ashes (%) | 4.45 | 10.05 |
| Protein (%) | 16.51 | 13.47 |
